# The governance gap: Determinants of health, international humanitarian law violations, and the case for health diplomacy

**DOI:** 10.1371/journal.pgph.0007482

**Published:** 2026-10-13

**Authors:** Ellen Beer, José Chen-Xu, Regina Yuen, Andrea Planatscher, Rebecca Gilmour, Zara Ahmed, Cristina Santamaria-Plaza, Nunzio Zotti, Milena Vasic, Mariam Hachem, Francesco Rosiello, Duha Shellah

**Affiliations:** 1 Public Health in Emergencies and Disasters Working Group, World Federation of Public Health Associations, Geneva, Switzerland; 2 Karolinska Institutet, Stockholm, Sweden; 3 Comprehensive Health Research Centre (CHRC), Public Health Research Center, LA-REAL, National School of Public Health, NOVA University of Lisbon, Lisbon, Portugal; 4 ISGlobal, Barcelona, Spain; PLOS: Public Library of Science, UNITED STATES OF AMERICA

*“Public health is and has always been about politics informed by evidence. We are agents of change for society and as such we are in the political arena”* [1].

## Introduction

Twelve years after the Lancet-Oslo University Commission on Global Governance for Health [2] global governance frameworks for protecting health in conflict continue to be treated as separate humanitarian, geopolitical, and security issues rather than an integrated part of everyday public health governance. We sit in a time of record attacks on healthcare and severe consequences of armed conflict. In 2024, over 3,600 attacks on healthcare were recorded globally, a 15% increase from 2023, and 62% increase from 2022 [3]. In 2024, over 900 healthcare workers were killed, 470 detained, and 140 kidnapped, and more than 1,100 health facilities were destroyed amid systematic obstruction of ambulances and humanitarian access [4]. In the last year fatal attacks on humanitarian personnel were reported in the Democratic Republic of Congo, Gaza and the West Bank, Lebanon, Myanmar, South Sudan, Sudan, and Ukraine [4].

Global governance for health must explicitly govern the political, commercial, environmental, and epistemic determinants of health at an institutional level and governance to protect life in conflict should be part of routine public health governance discourse, with health diplomacy responding more directly to upstream drivers of health impacts and health system collapse, and IHL violations.

### IHL violations as a governance issue

IHL, established in 1949 through the Geneva Conventions, aims to limit the impacts of conflict, protect civilians, health systems, and the environment, by restricting the methods of warfare [5]. Attacks on healthcare, humanitarian aid restrictions, and destruction of civilian infrastructure and the environment mark the point at which political, commercial, and environmental determinants of health produce their most system-destroying consequences. Political determinants or decisions shape whether populations are exposed to violence, deprivation, and displacement while commercial and regulatory decisions shape the circulation of resources, including weapons.

Commercial actors shape population health in ways that require active governance, such as binding regulation of tobacco marketing/sales, conflict-of-interest safeguards limiting industry influence on policy. Outside of international arms trade treaties, everyday policies governing the individual and institutional relationship with the arms sector are less ubiquitous, despite its relationship to mortality, displacement and environmental harm [6]. As a structural, upstream driver of IHL violations, the arms sector warrants similar governance standards. If public health has established norms requiring disclosure and management of tobacco industry links across publications, funding, and employment, should comparable norms not apply to institutional relationships with the arms sector?

### Environmental determinants of health

Environmental harm in conflict further demonstrates why this is of direct relevance to governance discourse, advocacy and action in planetary health. Armed conflict contaminates agricultural land, water systems, and air, which significantly threatens health through chronic disease, malnutrition, and intergenerational trauma. Attacks on water infrastructure contribute to cholera outbreaks, while carbon emissions from military operations exceed annual emissions of entire countries [7]. Environmental violations hold enduring consequences of conflict and reinforce a cycle in which ecological degradation undermines population health, weakens resilience, and increases vulnerability to further conflict.

The health consequences of the use of Agent Orange in Vietnam and unexploded ordnance in Laos and Cambodia remain visible today [8]. Such events prompted Additional Protocol I that prohibits warfare causing “*widespread, long-term and severe damage to the natural environment*” and treaties such as the 2008 Convention on Cluster Munitions. Only signatory states are obliged to comply. Governing the environmental determinants through commercial and political routes must be a priority within planetary health.

### Advocacy and decolonisation

Failure to govern conflict-related determinants of health disproportionately affects populations already structurally marginalised within health systems. Women, children, Indigenous peoples, gender-diverse people, older adults, people with chronic conditions and disabilities, and displaced people experience the most severe consequences of armed conflict and IHL violations [9]. Advocacy for marginalised population health is therefore inextricably linked to advocacy for evolution of governance.

Decolonising global health scholarship highlights how power asymmetries persist in public health in agenda-setting, funding allocation, authorship, representation, and control over knowledge production, and indeed which determinants and commercial sectors are governed [10]. The devaluation of local knowledge, lived experience, and political analysis constitutes violence and helps enable selective recognition of IHL violations [11,12].

In conflict-affected settings, this can explain whose suffering is recognised, whose security is prioritised, which crises attract diplomacy, and whose knowledge is considered authoritative in humanitarian health research, advocacy, and policy. Decolonising public health requires examining how coloniality persists through governance structures, shaping who is protected.

### Health diplomacy as a governance mechanism

Recognising IHL violations as governance failures requires practical mechanisms through which public health actors can engage politically and internationally. Health diplomacy has evolved beyond soft power [13] into a means for managing crises, fostering trust, and negotiating cooperative action during challenging times. It provides a tool for engaging with political determinants that lie upstream of IHL violations. In a turbulent world, health diplomacy promotes coordination and cooperation to enhance health outcomes [14], and provides a neutral platform to unblock traditional political channels, bringing the focus onto health. This allows cross-border coordination on issues such as water and energy security, vector control, and health system protection in fragile contexts [15]. Thus, strengthened health diplomacy can function as a response tool and a preventive one, by reducing risk, building trust, and supporting more resilient recovery pathways. Given the role of health diplomacy in international security, it is increasingly important to equip public health governance actors with the knowledge and skills to effectively integrate a health lens into international conflict policies, dialogues, and negotiations.

## Conclusion

IHL violations represent failures in governing political, commercial, environmental, and epistemic determinants of health. Preventing disproportionate health and health-system impacts from conflict and IHL violations requires stronger governance of commercial, political, and environmental determinants of health, as part of broader global governance for health. This requires addressing power asymmetries in agenda-setting, knowledge production, and decision making. Health diplomacy, supported by training in governance, negotiation, and conflict-aware health policy, can help operationalise this agenda.
